# Regional Hereditary Cancer Program in Chile: A scalable model of genetic counseling and molecular diagnosis to improve clinical outcomes for patients with hereditary cancer across Latin America

**DOI:** 10.1186/s40659-024-00579-x

**Published:** 2024-12-23

**Authors:** Natalia Landeros, Laura Vargas-Roig, Silvina Denita, Alejandra Mampel, Rafael Hasbún, Hernán Araya, Iván Castillo, Camila Valdes, Marcela Flores, Juan Salgado Salter, Katherin Vasquez, Jacqueline Romero, Ramón Pérez-Castro

**Affiliations:** 1 Unidad de Innovación en Prevención y Oncología de Precisión Centro Oncológico, Facultad de Medicina, Unidad de Innovación en Prevención y Oncología de Precisión Universidad Católica del Maule, Talca, 3480094 Chile; 2 In Vivo Tumor Biology Research Facility, Centro Oncológico, Facultad de Medicina, Universidad Católica del Maule, Talca, 3480094 Chile; 3 Biomedical Research Labs, Facultad de Medicina, Universidad Católica del Maule, Talca, 3480094 Chile; 4 Tumor Biology Laboratory, Institute of Medicine and Experimental Biology of Cuyo, National Research Council of Argentine, Mendoza, Argentina; 5 Medical School, National University of Cuyo, Mendoza, Argentina; 6 HEMA SAS, Mendoza, Argentina; 7 University Hospital, Mendoza, Argentina; 8 Hospital Regional de Talca (HRT), Talca, 3480094 Chile

**Keywords:** Breast cancer, Pathogenic variations, Multigene panel testing, Cancer genetic counseling, Variants of uncertain significance

## Abstract

**Background:**

Breast cancer is a leading cause of cancer-related mortality worldwide, with hereditary forms accounting for approximately 10% of cases. In Chile, significant gaps exist in genetic counseling and testing, particularly within the public health system. This study presents the implementation and outcomes of the first regional hereditary cancer program in the Maule region of Chile, aimed at improving detection and management of hereditary breast cancer.

**Methods:**

A cohort of 48 high-risk breast cancer patients from the Hospital Regional de Talca received genetic counseling and underwent Next-Generation Sequencing multigene panel testing. The program was established through collaboration between multiple institutions, leveraging telemedicine and outsourcing sequencing analysis to address regional gaps.

**Results:**

Pathogenic or likely pathogenic variants were identified in 12% of patients, including in *BRCA1*, *BRCA2*, *TP53*, and *PALB2*. Notably, novel pathogenic variants in *BRCA1* (rs80357505) and *TP53* (rs1131691022) were discovered, highlighting the unique genetic landscape of the Chilean population. Additionally, 70 variants of uncertain significance were found across 42 genes, particularly in *FAN1*, *MSH6*, and *FANCI*, underscoring the need for further research. The program’s collaborative approach effectively bridged critical gaps in genetic services, providing high-quality care within the public health system despite limited resources.

**Conclusions:**

The Regional Hereditary Cancer Program addresses significant gaps in genetic counseling and testing in Chile’s public health system. This scalable model enhances early detection and personalized treatment for hereditary cancer patients and could be adapted to other regions across Latin America.

## Background

Breast cancer (BC) is the leading cause of cancer death among women worldwide, and the most frequently diagnosed cancer [1]. BC has a high worldwide incidence, as reflected by the 2020 data showing 2,261,419 new cases (11,7% of all cancers) and 684,996 deaths (6.9% of all cancer-related deaths) [2]. In Chile, GLOBOCAN estimated 5,331 new cases of BC for the year 2020. This cancer is also the leading cause of cancer death among Chilean women, with 1,674 deaths associated with this diagnosis [2].

BC is considered a complex, heterogeneous, and multifactorial disease, arising from the accumulation of multiple genetic alterations [3, 4]. Approximately 10% of BC cases are hereditary, associated with germline pathogenic variants in BC susceptibility genes [5, 6]. Additionally, it is predicted that an additional 20% of BC cases have a familial genetic component. Therefore, the predisposition to BC is not attributable to a variant in one single inherited gene of high penetrance but to variants in genes of moderate penetrance and polygenic inheritance [7–9].

As the cost-effectiveness of sequencing technology advances, there is a growing focus on research into the genomic origins of cancer. The use of multi-gene panel testing for hereditary cancer risk assessment is becoming increasingly important in clinical practice. Multigene panels should include high penetrance genes such as *BRCA1*, *BRCA2*, *TP53*, *PTEN*, *STK11*, *CDH1*, and *PALB2* as well as low to moderate penetrance genes such as *ATM*, *CHEK2*, *NF1*, *RAD50*, among others. However, the clinical significance of some of these genes is not yet fully defined [10, 11]. Genetic determination of the hereditary cause of the diagnosis can guide surgical management and other treatments.

The National Comprehensive Cancer Network (NCCN) suggested that multigene panel testing should be offered broadly systematically in the context of professional genetic expertise with counseling developed pre- and post-testing [12]. Both presence and absence of pathogenic/likely pathogenic variants have implications for patients diagnosed with hereditary cancer [7]. Identifying individuals with pathogenic or likely pathogenic variants in a heritable BC-associated gene provides the opportunity to manage patients at increased cancer risk through early detection, chemoprevention, and risk-reducing surgeries. However, effective identification of mutated high-risk cancer genes such as *BRCA1* and *BRCA2* depends on the availability of and access to genetic counseling and testing for people at high risk of a hereditary cancer syndrome.

Despite these advancements, less than 20% of individuals with a family history of BC who meet hereditary cancer risk criteria have access to genetic counseling or interventions that could reduce mortality [13]. In Chile, there is a lack of comprehensive national programs for cancer risk assessment and genetic counseling. The National Cancer Plan established by the Ministry of Health in 2022 emphasizes cancer prevention strategies, including the identification of high-risk individuals and the provision of oncological genetic counseling [14]. However, significant gaps remain in the availability of specialized professionals, appropriate infrastructure, and access to genetic testing services, particularly for 80% of the population enrolled in the public health system. This highlights the need for more inclusive programs that can bridge this gap and ensure equitable access to these crucial services [15, 16].

Patients with a family history of BC, another primary tumor, bilateral BC, or early-onset BC require a counseling consultation to review their multigenerational pedigree, collect clinical history, and be referred for molecular genetic testing to determine if a hereditary cancer syndrome is present. A second counseling consultation is necessary following the molecular genetic test to communicate the results and additional recommendations for cancer monitoring, local therapy options, risk-reducing interventions, and identification of family members who may benefit from the test [17, 18].

In response to these needs, a regional comprehensive cancer risk assessment and genetic counseling program was implemented in the Maule region, aimed at decentralizing access to genetic counseling and molecular diagnosis for oncology patients. This program involves collaboration between multiple national and international institutions, providing universal, free, high-quality access to genetic counseling and Next-generation sequencing (NGS) multigene panel testing to identify germline pathogenic variants in high-susceptibility BC genes. This approach could reallocate public resources from treatment to prevention and ultimately reduce deaths due to hereditary-familial cancer. This study aims to characterize genetic variants associated with hereditary BC in a high-risk population and evaluate the implementation of the first regional comprehensive cancer risk assessment and genetic counseling program within Chile, specifically in the Maule region. By addressing the gaps in access to genetic testing and oncological counseling, this first Chilean regional hereditary cancer program sets a precedent that could be adapted for other malignancies and regions. By providing a scalable model, this program has the potential to significantly improve outcomes for hereditary cancer patients across Chile and in other Latin American countries with similar infrastructure and resource constraints.

## Methods

### Breast cancer population study

Patients with suspected hereditary BC syndrome were recruited from the Hospital Regional Talca, which cares for cancer patients from the entire Maule Region. The patients were referred for oncology counseling by telemedicine and molecular genetics study (analysis of the multigene panel of 162 genes associated with hereditary cancer). In the counseling consultation, clinical, pathological and family information was obtained with which the patient’s genealogy was generated, incorporating healthy and cancer patients in the family. This research was approved by the institutional ethics committee of Universidad Católica del Maule (176/2021). All participants gave their written informed consent and completed a questionnaire related to their medical and reproductive history, ethnicity, and risk factors. In total, 48 cases with BC were selected for this pilot study.

Syndrome-specific indications were included such as: (i) families with BC and ovarian cancer, tubal carcinoma, or primary peritoneal carcinoma regardless of the age of onset, (ii) families with BC, endometrial cancer, and/or thyroid carcinoma, (iii) families with BC and pediatric tumors (such as sarcomas, leukemias and central nervous system tumors), and (iv) families with BC and gastrointestinal disease (such as hamartomatous polyps, diffuse gastric cancer, colon cancer).

### Sampling and DNA isolation

The blood sample was extracted into a tube with EDTA anticoagulant and taken to the Biomedical Research Laboratories of the Universidad Católica del Maule for processing it. Genomic DNA was extracted from peripheral blood leukocytes using E.N.Z.A Blood DNA Maxi Kit, Omega Bio-Tek (D2492-03) according to the manufacturer’s instructions and was quantified using NanoDrop™ 2000.

### Genes analyzed and library preparation by NGS sequencing

The sequencing was made in Sistemas Genómicos - Spain. The 162 genes OncoClever-GeneSGKit library was used. For the library preparation ‘Paired end’ reads 101 nt length were generated. Then, targeted regions were enriched using targeted sequencing protocol and sequenced in an Illumina NextSeq sequencing platform. Reads were aligned against the human reference genome version GRCh38/hg38. Read alignment was performed using BWA and ‘in-house’ scripts.

Genes included in the library were: *ACD, BMPR1A,, DDB2, ERCC4, FANCM, HNF1B, MET, NF2, PMS2CL, RAD51C, SDHAF2, TERC, WRN, AIP, BRCA1, DICER1, ERCC5, FH, HOXB13, MITF, NFIX, POLD1, RAD51D, SDHB, TERT, WT1, AKT1, BRCA2, DIS3L2, ERCC6, FLCN, HRAS, MLH1, NHP2, POLE, RB1, SDHC, TGFBR2, XPA, ALK, BRIP1, DKC1, FAN1, G6PC3, IDH1, MLH3, NOP10, POLH, RECQL, SDHD, TINF2, XPC, APC, BUB1, DLST, FANCA, GALNT12, JAGN1, MNX1, NSD1, POT1, RECQL4, SLC25A11, TMEM127, XRCC2, AR, CDC73, EGLN1, FANCB, GCM2, KIF1B, MRE11, NTHL1, PRCC, RET, SLX4, TP53, XRCC3, ATM, CDH1, EGLN2, FANCC, GDNF, KIT, MSH2, PALB2, PRKAR1A, RFWD3, SMAD4, TSC1, ATR, CDK4, ELANE, FANCD2, GFI1, LZTR1, MSH3, PARN, PRSS1, RNF139, SMARCB1, TSC2, ATRX, CDKN1B, EPAS1, FANCE, GPC3, MAD2L2, MSH6, PDGFRA, PTCH1, RNF43, SMARCA4, UBE2T, AXIN2, CDKN2A, EPCAM, FANCF, GPR101, MAX, MSR1, PHOX2B, PTCH2, RPS20, SMARCE1, VHL, BAP1, CHEK2, ERCC1, FANCG, GREM1, MC1R, MUTYH, PIK3CA, PTEN, RTEL1, SRP54, VPS45, BARD1, CSF3R, ERCC2, FANCI, HAX1, MDH2, NBN, PMS1, RAD50, SCG5, STK11, WAS, BLM, CTNNA1, ERCC3, FANCL, HNF1A, MEN1, NF1, PMS2, RAD51, SDHA, SUFU, and WRAP53.*

### Interpretation of identified variants

Germline variants with allele frequency < 0.02 based on allele frequencies and coverage > 20 reads were selected. The variants found were classified according to the American College of Medical Genetics and Genomics (ACMG) guidelines as pathogenic, likely pathogenic, benign, likely benign or variants of unknown significance [19]. Variants were described according to the nomenclature recommendations of the Human Genome Variation Society [20].

### Validation and segregation analysis by Sanger sequencing

To validate the clinically relevant variants (pathogenic and likely pathogenic) Sanger sequencing was performed. In addition, the identified pathogenic variants were studied by Sanger in first-degree relatives. The primers used for sequencing are detailed in Table 1.

**Table 1:** List of PCR primers used to validate mutations by Sanger sequencing

Gene	Primer forward	Sequence 5’->3’	Primer reverse	Sequence 5’->3’	PCR product size
*BRCA1*	rs80357208-F	AGGATGAAGAGCTTCCCTGC	rs80357208-R	GACCAACTCCCTGGCTTTCA	340 bp
	rs80357505-F	TGACTCACATGATGGGGAGTC	rs80357505-R	ACCATTCTGCTCCGTTTGGT	440 bp
*BRCA2*	rs11571658-F	ACGAACATTCAGACCAGCTCA	rs11571658-R	GGTGAAGCCTGTTCTTTTCCC	505 bp
*PALB2*	rs876659571-F	ACTGAGTCCTAAACGCATGGA	rs876659571-R	TGTTTATGCAGCTCCTGGCA	450 bp
*TP53*	rs1131691022-F	AGGTACTTGAAGTGCAGTTTCT	rs1131691022-R	TGTGGTTATAGGATTCAACCGGA	420 bp

bp: Base pairs

### Detection of large genomic rearrangement using copy number variation analysis

The algorithm for the detection of copy number variants (CNVs) using NGS (multigene panel) is based on the comparison of the reading depth of a certain region of the genome of the sample under study in comparison with a set of samples used as a reference [21]. The underlying hypothesis of this method is that the read depth of a genomic region is correlated with the number of copies of that region. CNVs were evaluated for the genes *ATM*, *BARD1*, *BLM*, *BRCA1*, *BRCA2*, *BRIP1*, *CDH1*, *CHEK2*, *EPCAM*, *ERCC4*, *FANCM*, *MLH1*, *MSH2*, *MSH6*, *NBN*,*NF1*, *PALB2*, *PMS2*, *PTEN*, *RAD50*, *RAD51*, *RAD51C*, *RAD51D*, *RECQL*, *STK11*, *TP53*, *XRCC2*, and *XRCC3.*

## Results

**Fig. 1:**
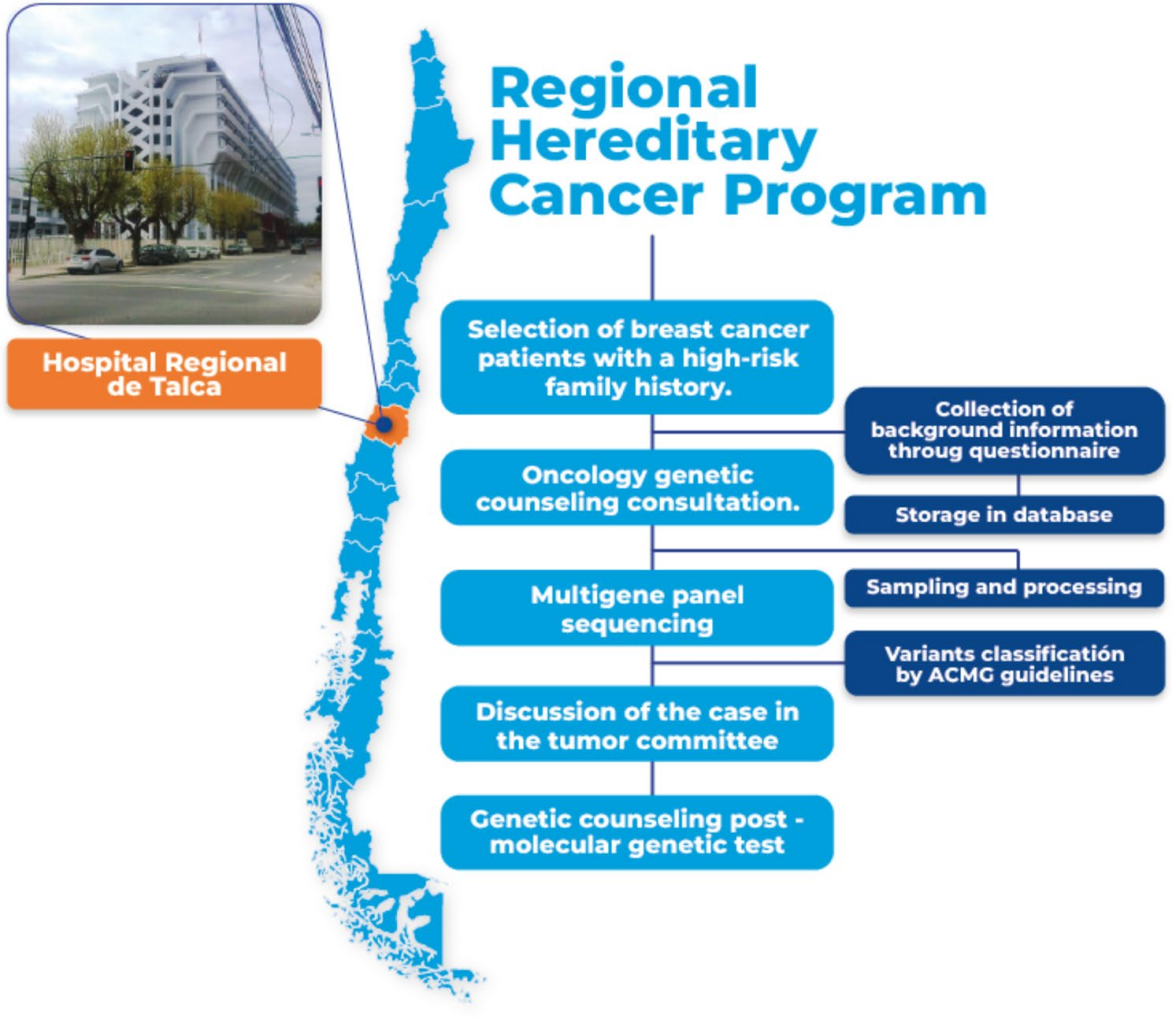
Workflow of the Regional Hereditary Cancer Program implemented for Hospital Regional de Talca of the Maule Region. The main map of Chile indicates the location of the Maule Region where the program is implemented. The program compiles detailed information on each patient recruited into the Hereditary Cancer Program into an exclusive database. This information includes personal history, clinical history of pathological anatomy, and genetic counseling details. The latter encompasses patient identification, age, sex, age at diagnosis, type of cancer, histology, molecular subtype, presence of first-degree and second-degree relatives, among other data. Additionally, the database records the variants identified through genetic testing

The workflow designed for the Regional Hereditary Cancer Program, implemented at the Hospital Regional de Talca, is shown in Fig. 1. This program was initially based on the framework of hereditary BC due to its well-established criteria and significant impact on public health. Cases of familial and hereditary BC typically present clinical characteristics that raise suspicion, necessitating a thorough evaluation in clinical practice. International criteria were applied to refer patients for oncological genetic counseling, including BC diagnosed before the age of 50, bilateral or multicentric BC (synchronous or metachronous), BC in men, BC in high-risk ethnic groups (such as Ashkenazi Jewish), two or more cases of BC in first- and second-degree relatives, individuals with BC and another primary tumor, and triple-negative BC diagnosed before the age of 60 [23].

These patients were referred from the Hospital Regional de Talca for an oncological genetic counseling consultation by telemedicine, in which clinical, personal, and family history of individuals are collected, with the aim of identifying patients who require a genetic-molecular study. Then these patients are scheduled for blood sample collection, which is processed (DNA extraction) and sent for analysis by sequencing of a multigene panel associated with hereditary cancer. The variants found in the genetic-molecular study are classified according to the ACMG categories [19]. The results are summarized in a genetic study results report that includes pathogenic, likely pathogenic, and variants of uncertain significance. Recommendations for follow-up and risk reduction surgeries are also included, according to international NCCN criteria.

For this program, a multidisciplinary tumor committee was formed (made up of oncologists, mastologists, midwives, molecular biologists and geneticists). Finally, the results are delivered to the patients in a counseling consultation following the genetic-molecular study.

### Demographic and clinical features of the patients

For the implementation of the Regional Hereditary Cancer Program, we selected a group of 48 probands with BC, between June 2022 and June 2023, who were recruited at the Hospital Regional de Talca, Chile. Patients were referred for genetic counseling consultation and subsequent genetic-molecular study. The majority of people analyzed were women (91.6% *n* = 44), while only 8.3% (*n* = 4) were men with BC. 63% (30/48) had at least one first- or second-degree relative with a history of some type of cancer. The average age at diagnosis was 42 years (range 25 to 80 years), with 77% (*n* = 37) of the recruited patients having a diagnosis age of less than 50 years, 5 patients had bilateral BC, and 1 patient had BC and other primary tumor. Eight patients had triple negative BC (lack of estrogen receptor, progesterone receptor, and human epidermal growth receptor 2). The demographic and main clinical characteristics of the program patients are summarized in Table 2. Interestingly, 11 of the 44 women in the study (25%) reported that they had 1 or more spontaneous abortions.

**Table 2:** Demographic and clinical characteristics of the patients recruited in the Regional Hereditary Cancer Program

		Number of patients	%	
Total individuals	Female	44	91,6	
	Male	4	8,3	
Age at diagnosis (years)	20–29	6	12,5	
	30–39	16	33,3	
	40–50	15	31,3	
	51–80	11	22,9	
Cancer Family History	one relative	11	22,9	
	≥ two relatives	19	39,5	
Bilateral BC		6	12,5	
TNBC		6	12,5	
BC and another primary tumor		4	8,3	
one or more spontaneous abortions		11	22,9	

BC: breast cancer, TNBC: triple negative breast cancer

### Variants found through a multigene panel of genes associated with hereditary cancer

A total of 48 patients with a high-risk history for BC were recruited for the establishment of the Regional Hereditary Cancer Program at the Hospital Regional de Talca. Six patients with pathogenic/likely pathogenic variants were detected, which are summarized in Table 3. We detected a 12% of individuals carrying variants. These variants were located in the high-risk genes *BRCA1*, *BRCA2*, *TP53*, and *PALB2*. The *BRCA1* p.Gln1273Ter variant was found in two unrelated families.

**Table 3:** Pathogenic or likely pathogenic variants detected in the cancer susceptibility genes in a high-risk population

Gene	Transcript Ref. seq.	HGVS Nucleotide change	HGVS Amino acid change	dbSNP rsID	Number of carriers	Age at diagnosis	Family history of cancer
*BRCA1*	NM_007300.4	c.3817 C > T	p.Gln1273Ter	rs80357208	2	45 53/62	1 relative with BC; Bilateral BC and 3 relatives with BC
		c.1439dup	p.Asn480LysfsTer10	rs80357505	1	37/37	Bilateral BC
*BRCA2*	NM_000059.4	c.6275_6276del	p.Leu2092Profs*7	rs11571658	1	25	1 relative with BC
*PALB2*	NM_024675.4	c.2288_2291del	p.Leu763Ter	rs876659571	1	34	Man with BC and 1 relative with BC
*TP53*	NM_000314.8	c.1024del	p.Arg342GlufsTer3	rs1131691022	1	36	1 relative with BC

*Abbreviations* HGVS: Human Genome Variation Society, BC: breast cancer

Using the algorithms for the identification of possible copy number variants (CNVs) with the genomic information from the multigene panel sequencing, no pathogenic or likely pathogenic variants were identified in the genes  *ATM*, *BARD1*, *BLM*, *BRCA1*, *BRCA2*, *BRIP1*, *CDH1*, *CHEK2*, *EPCAM*, *ERCC4*, *FANCM*,*MLH1*, *MSH2*, *MSH6*, *NBN*, *NF1*, *PALB2*, *PMS2*, *PTEN*, *RAD50*, *RAD51*, *RAD51C*, *RAD51D*, *RECQL*, *STK11*, *TP53*, *XRCC2*, and *XRCC3.*

### Characteristics of patients carrying germline pathogenic variants and their relatives

**Fig. 2:**
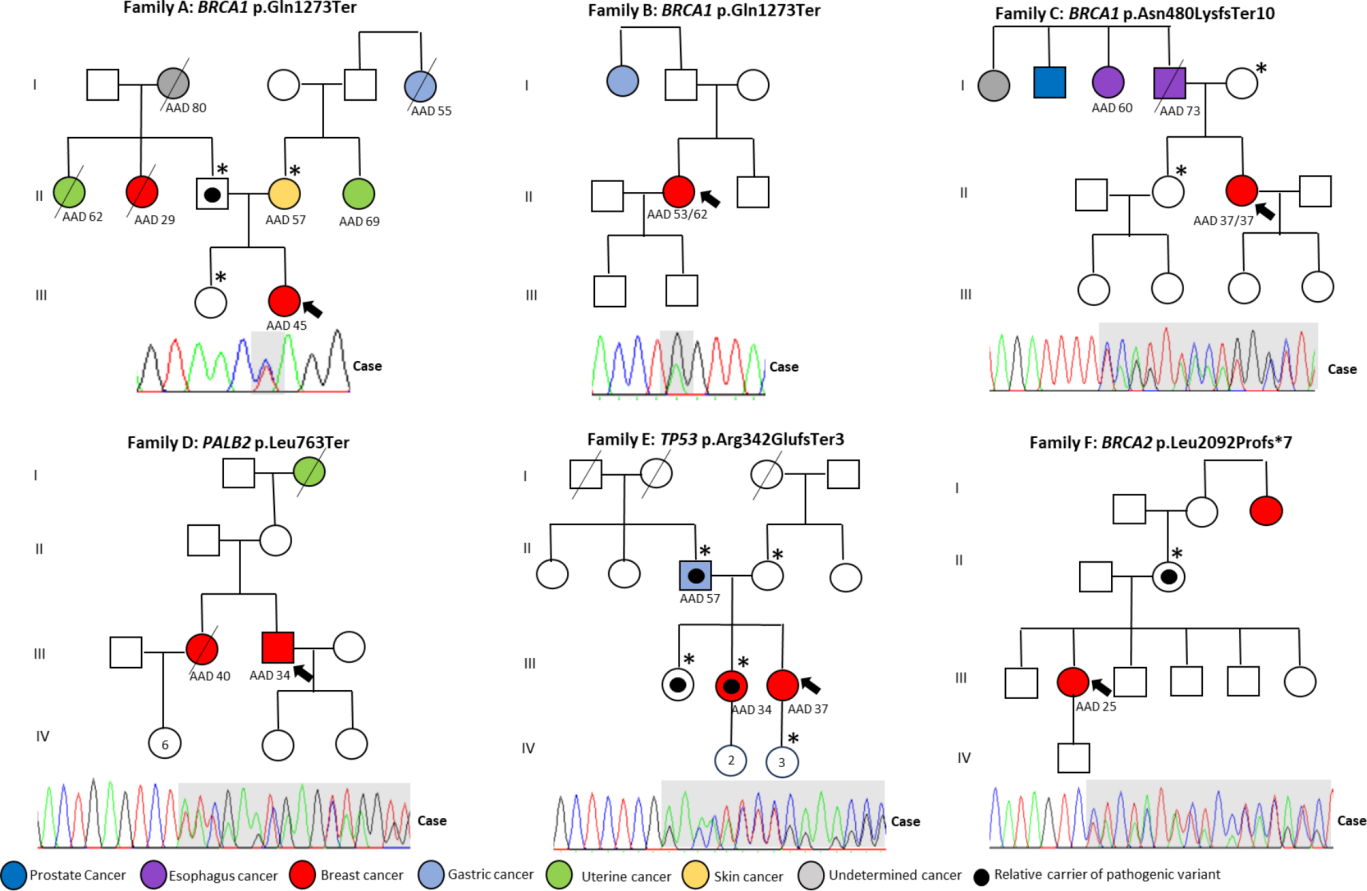
Genealogy of the families of patients with germline pathogenic variants. The age of cancer diagnosis is reported for each individual, the arrow indicates the proband of each family, * indicates family members who were evaluated for the pathogenic mutation. The pathogenic variants were confirmed by Sanger (gray in electropherograms), all identified in a heterozygous state. The age of cancer diagnosis (AAD)

Once patients carrying pathogenic variants have been identified, their first-degree relatives are invited to participate in the study to determine if they carry the specific pathogenic variant through Sanger sequencing. Figure 2 shows the pedigrees of the 6 patients carrying monoallelic pathogenic variants identified as part of the program. The co-segregation of the pathogenic variants was carried out in four families. Patients and their family members who were carriers were offered follow-up measures and/or risk reduction surgeries according to the international NCCN criteria and the specific variants they carried. In Family A, the healthy father is a carrier of the pathogenic variant, but the only sister of the index case is not a carrier. The index case had previously undergone a total mastectomy (MT) before the genetic study, and after genetic counseling, a prophylactic MT of the other breast and oophorectomy were suggested. In Family C, the only sister of the index case is not a carrier of the pathogenic variant in the *BRCA1* gene. The index case initially had a surgical plan for a right total MT and left prophylactic MT, which was changed to a bilateral total mastectomy and bilateral adnexectomy after the genetic study. In Family D, the index case had received a left total MT and right adenomastectomy before the genetic study, and their relatives did not agree to cascade genetic testing. In Family E, the father of the index case, who has gastric cancer, and the two sisters, one with BC, are carriers of the pathogenic variant in the *TP53* gene. In Family F, the healthy mother of the index case is a carrier of the pathogenic variant in the *BRCA2* gene. The index case’s surgical plan was changed from a total MT to a bilateral MT and oophorectomy.

The identification of pathogenic variants in program patients has significantly impacted their treatment by providing personalized risk reduction measures to prevent secondary cancers. Additionally, long-term follow-up and continuous monitoring of patients and their carrier relatives are crucial for early detection, preventive strategies, and tailored therapeutic adjustments, thereby improving clinical outcomes and patient quality of life.

**Fig. 3:**
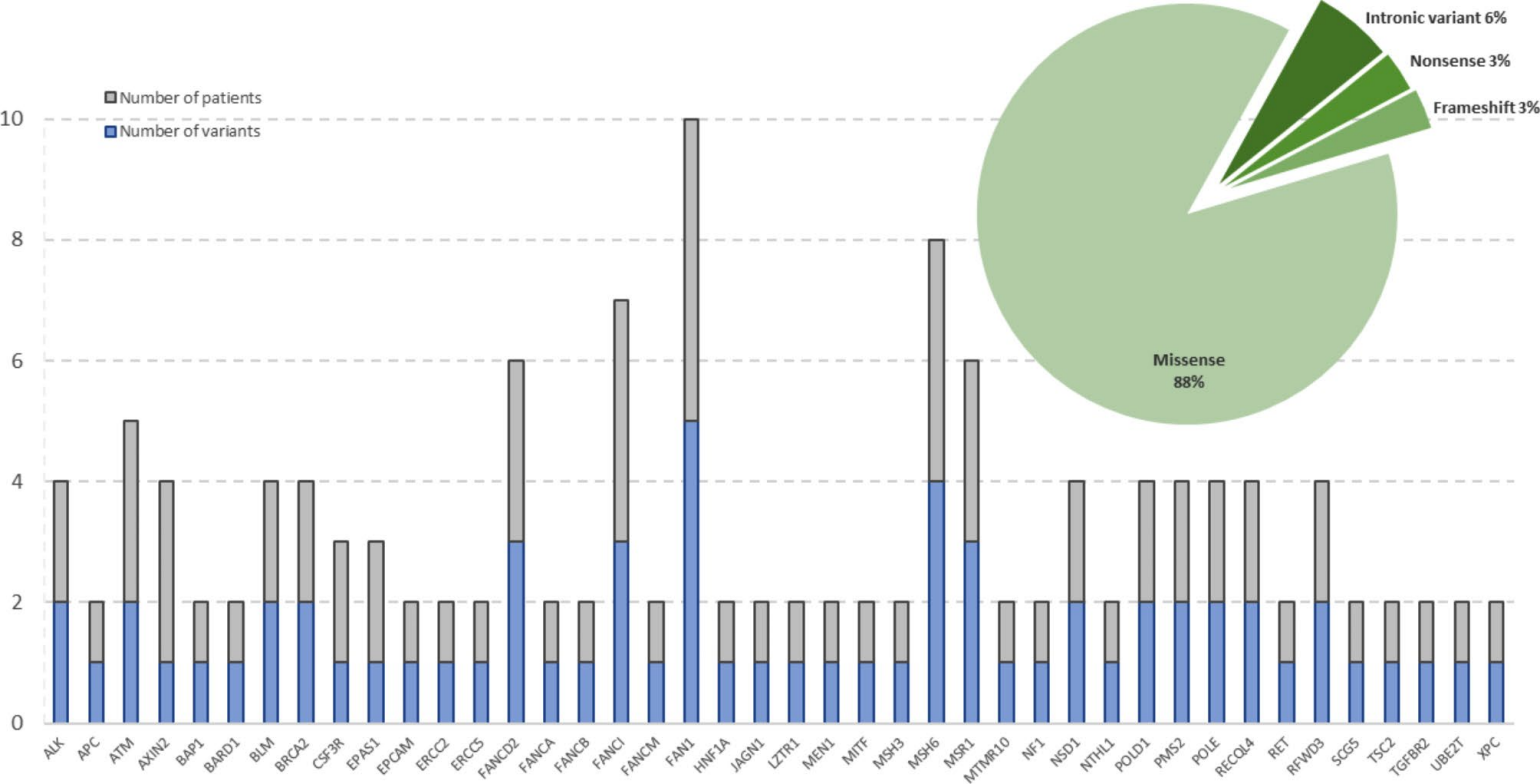
Results of the variants of uncertain significance identified in the 162 genes studied. **a**) Bar graph representing the number of VUS found in each gene and the number of patients carrying these VUS, **b**) The pie graph representing the types of variants classified as uncertain significance

Variants of uncertain clinical significance (VUS) according to the ACMG (American College of Medical Genetics) rules are those where it is not possible to define their causal relationship with the development of pathology, in this case with BC [19]. A total of 70 VUS were classified into 42 genes associated with hereditary cancer (Fig. 3), which corresponded mainly to missense variants (62/70), 4 intronic variants, 2 nonsense variants, and 2 small indels. The genes that presented the highest number of VUS were *FAN1* (*n* = 5), *MSH6* (*n* = 4), *FANCI* (*n* = 3), and *FANCD2* (*n* = 3). Among the 48 patients we analyzed, we found 37 of them had at least one VUS, with some individuals having up to five VUS. This gives us an average of two VUS per individual in our study.

### Identification of single nucleotide variants in the multigene panel for hereditary cancer

In addition to high- and moderate-risk genes, genome-wide association studies have identified common, low-risk single nucleotide variants (SNVs) associated with BC. These typically confer a 1.05- to 1.50-fold increase in risk. While these SNVs do not individually offer significant insight into a person’s risk of developing BC, their combined effect provides a degree of risk discrimination. This is valuable for population-wide prevention and early detection programs [7, 24, 25]. Furthermore, they provide insight into the frequent SNVs present in Chilean BC patients. We identified a total of 18 SNVs in the multigene panel analyzed. In the literature, these SNVs are reported to be associated with BC and other cancers. We summarized the SNVs in the main gene associated with BC in Table 4.

**Table 4:** Description of the single nucleotide variant found in the multigenic panel

Gene	HGVS Nucleotide change	HGVS Amino acid change	dbSNP rsID	VAF	Association with cancer	Ref
*AXIN2*	c.148 C > T	p.Pro50Ser	rs2240308	T: 0,322	BC, CCR, PC	[26–29]
*BRCA1*	c.3113 A > G	p.Glu1038Gly	rs16941	G: 0,375	General cancer risk	[30]
	c.3548 A > G	p.Lys1183Arg	rs16942	G: 0,354	BC	[31, 32]
*BRCA2*	c.2971 A > G	p.Asn991Asp	rs1799944	G: 0,156	BC	[33, 34]
	c.7242 A > G	p.Ser2414=	rs1799955	G: 0,062	BC	[35]
	c.1114 A > C	p.Asn372His	rs144848	C: 0,322	BC, NHL	[36, 37]
*CDH1*	c.48 + 6 C > T	**-**	rs3743674	T: 0,656	BC	[38]
*EPCAM*	c.428T > C	p.Met143Thr	rs1126497	C: 0,458	BC, GC, CC	[39–41]
*MSH3*	c.3133G > A	p.Ala1045Thr	rs26279	A: 0,729	BC, CCR	[42, 43]
*PIK3CA*	c.1173 A > G	p.Ile391Met	rs2230461	G: 0,083	BC	[44]
*WRN / RECQL2*	c.4099T > C	p.Cys1367Arg	rs1346044	C: 0,145	BC	[45]
*TP53*	c.215 C > G	p.Pro72Arg	rs1042522	G: 0,552	BC, AML, LC	[37, 46–48]

*Abbreviations* HGVS: Human Genome Variation Society; -: intronic variant without effect on the protein, VAF: variant allele frequency in 48 patients. BC: breast cancer, GC: gastric cancer, CCR: colorectal cancer, LC: lung cancer, AML: acute myeloid leukemia, PC: prostate cancer, CC: Cervical cancer, NHL: Non-Hodgkin lymphoma

## Discussion

The implementation of the Regional Hereditary Cancer Program in the Maule region represents a significant advancement in the identification and management of hereditary breast cancer patients. The detection of pathogenic variants in high-risk genes such as *BRCA1*, *BRCA2*, *TP53*, and *PALB2* in 12% of the studied patients underscores the critical need for genetic testing and counseling in populations with limited access to these services [19]. Notably, this program identified novel pathogenic variants, such as rs80357505 in *BRCA1* and rs1131691022 in *TP53*, which have not been previously reported in other populations. This discovery emphasizes the unique genetic landscape of the Chilean population and the importance of region-specific genetic research to better understand hereditary cancer risks in diverse populations.

A significant portion of the patients studied exhibited variants of uncertain significance, with a total of 70 VUS across 42 genes, highlighting the need for further research and functional studies to elucidate their clinical relevance [49]. In accordance with international guidelines, the VUS identified in our program’s database are subject to periodic review to ensure updates to their classification and to refine clinical recommendations accordingly. The genes *FAN1*, *MSH6*, and *FANCI* were among those with the highest number of VUS, complicating clinical decision-making due to the uncertain impact of these variants on hereditary cancer risk. This situation underscores the importance of continuous monitoring, reclassification efforts, and the development of updated guidelines to better manage these findings. The lack of functional assays for many VUS presents a considerable challenge, necessitating collaboration between clinical and research institutions to ensure accurate interpretation and appropriate patient management [49].

The program also successfully identified several SNVs associated with hereditary cancer. For example, the SNV rs2240308 in the *AXIN2* gene has been associated with an increased risk of BC, as well as colorectal and prostate cancers in various populations [26, 27]. In the *BRCA1* and *BRCA2* genes, SNVs such as rs16942 and rs1799944 have been linked to increased BC risk, further underscoring the importance of comprehensive genetic analysis in managing hereditary cancer syndromes [30, 33]. Additionally, the *TP53* gene variant rs1042522, identified in this cohort, is known to influence the susceptibility to various cancers, including BC and lung cancer, particularly in Asian and Caucasian populations [47, 48].

It is estimated that about 10 to 15% of all pregnancies end in spontaneous abortions [50]; in this group of clinically high-risk of hereditary BC patients, 22,9% of women were reported to have had one or more spontaneous abortions. A study carried out by Lambertini et al., in 1252 young women carrying a *BRCA* germline mutation, found a spontaneous birth rate of 10.3%, similar to that reported for the general population [51]. This finding suggests that while the presence of *BRCA* mutations may not significantly increase the risk of spontaneous abortions compared to the general population, it is crucial to consider other factors such as the impact of diagnostic screening procedures, cancer treatments, age, and overall health, which can influence reproductive outcomes in these women. The study highlights the importance of providing comprehensive reproductive counseling and support for women with hereditary BC to address their unique concerns and optimize their reproductive health outcomes.

An innovative aspect of this program is its approach to overcoming the lack of genetic counselors and technical capacities in the Maule region. By leveraging telemedicine for genetic counseling, outsourcing sequencing analysis to external laboratories, and establishing a multidisciplinary tumor board that operates across multiple institutions without requiring physical presence in the region, the program has successfully provided high-quality genetic services despite the region’s limited resources and capabilities [21, 22]. This model not only ensures that patients receive timely and accurate genetic assessments, but also serves as a scalable solution for other regions and countries facing similar challenges [12].

Moreover, the multidisciplinary tumor board established through this program plays a crucial role in personalized patient management. Comprising oncologists, mastologists, midwives, molecular biologists, and geneticists, the board has facilitated the implementation of tailored risk reduction strategies, including prophylactic surgeries such as mastectomies and oophorectomies, which have been pivotal in preventing secondary cancers in high-risk patients [11, 17]. The involvement of family members in genetic testing has also enabled the early identification of carriers among relatives, allowing for timely intervention and preventive measures in accordance with international guidelines, such as those from the National Comprehensive Cancer Network (NCCN) [23].

## Conclusions

The Regional Hereditary Cancer Program has significantly advanced the identification and management of patients with pathogenic variants linked to hereditary cancer syndromes. By facilitating personalized risk reduction strategies, including prophylactic surgeries, the program has effectively reduced the risk of recurrence and the development of new cancers in predisposed individuals. The program has also underscored the importance of long-term follow-up and continuous monitoring of patients and their carrier relatives, enhancing early detection, preventive measures, and tailored therapeutic interventions, ultimately improving clinical outcomes and patient quality of life.

The program’s success has led to its establishment at the Regional Hospital of Talca, including over 150 hereditary cancer patients to date. This model could be replicated in other regions to improve outcomes for hereditary cancer patients nationwide. Future efforts should focus on increasing awareness and participation in genetic testing, enhancing genetic counseling accessibility, and advancing research to identify additional genetic markers and effective preventive strategies, thereby continuing to improve the prognosis and quality of life for individuals at risk of hereditary cancers.

## Data Availability

All data generated or analysed during this study are included in this published article.

## Abbreviations

AAD: Age of Cancer Diagnosis
ACMG: American College of Medical Genetics and Genomics
AML: Acute Myeloid Leukemia
BC: Breast Cancer
CC: Cervical Cancer
CCR: Colorectal Cancer
CNVs: Copy number variants
GC: Gastric Cancer
HGVS: Human Genome Variation Society
LC: Lung cancer
MLPA: Multiplex Ligation Dependent Probe Amplification
MT: Mastectomy
NCCN: National Comprehensive Cancer Network
NHL: Non-Hodgkin lymphoma
PC: Prostate Cancer
SNVs: Single Nucleotide Variants
TNBC: Triple Negative Breast Cancer
VAF: Variant Allele Frequency
VUS: Variants of Uncertain Clinical Significance
